# The perceptions of pre-service and in-service teachers regarding a project-based STEM approach to teaching science

**DOI:** 10.1186/2193-1801-4-8

**Published:** 2015-01-05

**Authors:** Nyet Moi Siew, Nazir Amir, Chin Lu Chong

**Affiliations:** 13 Faculty of Psychology and Education, Education Block, Universiti Malaysia Sabah, Jalan UMS, 88400 Kota Kinabalu, Malaysia; 14 Greenview Secondary School, 15 Pasir Ris Street 21, Singapore, 518969 Singapore; 15 Faculty of Psychology and Education, Education Block, Universiti Malaysia Sabah, Jalan UMS, 88400 Kota Kinabalu, Malaysia

**Keywords:** Professional development, Project-based learning, Science, Teaching innovation, STEM

## Abstract

Whilst much attention has focused on project-based approaches to teaching Science, Technology, Engineering and Mathematics (STEM) subjects, little has been reported on the views of South-East Asian science teachers on project-based STEM approaches. Such knowledge could provide relevant information for education training institutions on how to influence innovative teaching of STEM subjects in schools. This article reports on a study that investigated the perceptions of 25 pre-service and 21 in-service Malaysian science teachers in adopting an interdisciplinary project-based STEM approach to teaching science. The teachers undertook an eight hour workshop which exposed them to different science-based STEM projects suitable for presenting science content in the Malaysian high school science syllabus. Data on teachers’ perceptions were captured through surveys, interviews, open-ended questions and classroom discussion before and at the end of the workshop. Study findings showed that STEM professional development workshops can provide insights into the support required for teachers to adopt innovative, effective, project-based STEM approaches to teaching science in their schools.

## Introduction

The Malaysian educational system is currently undergoing transformation, one emphasis of which is to create a generation who can think creatively, innovatively and critically (Ministry of Education 2012a). As part of the reform efforts, the Malaysian Ministry of Education (MOE) has created initiatives that aim to increase teachers’ and students’ competencies in Science, Technology, Engineering and Mathematics (STEM) subjects and create learning experiences that will prepare students for the considerable array of STEM career fields.

In spite of the emphasis on STEM, science and mathematics are not subjects of first choice for a majority of Malaysian high school students, whose interest in science subjects has been steadily falling (Phang et al. 2012). During the mid-1980s, the ratio of students taking science to arts subjects was 31:69. By 2012, this had fallen to 27:78 (Ministry of Education 2012b). The number of science stream students has dropped up to 29% since 2007 (Ayob 2012). One reported reason for falling enrolment in many science programs is that students are turned off by the way these subjects are taught, as Phang et al. (2012) reported that students find studying science to be difficult, boring and not worth the effort. These findings reflect broader concerns that declining interest of students in science and mathematics may stunt the efforts to improve technological innovations to make Malaysia a high income country by 2020.

Equally alarming, recent international comparisons of 15-year-old students’ performance in the 2012 Program for International Student Assessment (PISA) examination, indicate that Malaysian students scored below the international average, ranking 52 out of the 65 countries in mathematics, science and reading (OECD 2014). Further analysis shows that Malaysian students ranked 39 out of the 44 countries with a mean score of 422 on creative problem-solving, designed to assess students’ abilities to apply scientific and mathematical concepts to real-world problems (OECD 2014). The mean score for all countries was 500. Malaysia had more than half the share of low achievers, revealing students’ lack of the kind of skills needed to tackle real life problems that are increasingly needed in today’s workplace.

Malaysia plans to develop creative and innovative human capital to meet the nation’s need in the 21^st^ century. However, if current trends persist, the Science and Technology Human Capital Direction Plan 2020’s requirement of 60% STEM workforce may not be achieved. These trends raise the question ‘What can science educators do to enhance and promote interest in science and other disciplines in STEM?’

It follows that in order to attract more students into STEM careers, students should be provided with meaningful learning experiences that motivate and excite them, and that relate to their own context. To this end, science educators should be capable of offering learning experiences that engage students in realistic, thought-provoking problems, working with others, and applying their knowledge, skills and creativity in finding solutions to real-world problems. However, teachers may face significant challenges fostering an interest in STEM subjects. One of the biggest challenges for Malaysian primary and secondary science education is that few guidelines or models exist regarding how to teach using STEM approaches in the classroom. In view of these points, this study generated and examined a range of ideas on how to enhance awareness among teachers on science teaching through STEM approaches, and how these could motivate and excite students, as well as themselves, in teaching and learning science in schools.

### Literature review

#### STEM-project-based learning (STEM-PjBL) as pedagogy in the teaching and learning of science

Many scholars argue that for students to be fully prepared for careers in STEM, they should engage in pedagogical practices that reflect the interdisciplinary, ill-defined problems that scientists face (Anderson 2007; Clark and Ernst 2007; Marshall et al. 2007; Paige et al. 2008; Park-Rogers et al. 2007). In response, many countries have developed reforms and initiatives to shape teaching and learning across STEM disciplines. A number of these are interdisciplinary and integrative in nature, with blurred boundaries among the four disciplines (Wang et al. 2011). Linking STEM subjects offers considerable advantages, notably getting students to make sense of learning content across the four subjects, while promoting critical thinking and problem-solving skills (Beane 1997; Drake 1991; Drake 1998; Jacobs 1989; Miller 1995; Nielsen 1989).

To mirror scientists’ problem-based work, a carefully crafted interdisciplinary approach to STEM teaching allows students to experience ill-defined problems, and provide them with avenues to solve these through a variety of answers, as opposed to single answer solutions more typical of didactic teaching approaches. Very little research has explicitly examined project-based learning (PjBL) as a pedagogical framework for teaching interdisciplinary STEM subjects. Actually, many of the learning experiences advocated in STEM teaching approaches are similar to the underlying principles of PjBL. Hence, PjBL in STEM (or STEM-PjBL approach) has promise as a framework for STEM initiatives.

In a STEM-PjBL learning environment, important concepts in STEM subjects can be gained in solving complex problems (Hickey 2014). Teachers in this environment could guide students in small groups to develop a variety of solutions for a given problem, encouraging collaborative learning and strengthening critical thinking and communication skills (Hickey 2014). Moreover, as students engage with the STEM-PjBL approach, they are in essence mirroring the processes used by scientists and engineers to solve real-world issues through the active construction of new knowledge and the development of problem-solving skills (Flores et al. 2002; Gutstein 2003; Rogers and Portsmore 2004).

Design-Based Science (DBS) is one example of a STEM-PjBL approach. In DBS, students actively engage with problem solving through designing and building artefacts (Fortus et al. 2005). The DBS process provides students with opportunities to carry out series of experiments, with hands-on activities that relate to STEM subjects content (Satchwell and Loepp 2002). Research has also shown that project-based learning that integrates concepts in STEM subjects has enabled students to transfer their knowledge and skills to solve real-world problems (Fortus et al. 2005), which has in many occasions led to improved scores in higher-level mathematical problem solving and scientific process skills (Satchwell and Loepp 2002).

#### Motivating students through a STEM-PjBL approach

Educators have shown that the use of a STEM-PjBL approach has been useful in getting students to make sense of learning science content that is within the science syllabus students study in school. This approach has particularly worked well for the academically less-inclined students. Amir (2014) argues that while the prescribed science experiments in the secondary school activity books for these students allow teachers to engage them in learning through a hands-on approach, a large number of these experiments provide little room to foster and reward these students for being able to showcase their creativity through knowledge from science. Students, in doing these experiments, are seen to be going through a routine of steps as instructed by their teachers with hardly much opportunity for them to put their imaginative and inventive skills to good use (Abrahams and Millar 2008; and Abrahams and Millar 2008). Amir and Subramaniam (2014) mentioned that these students have been observed to ask questions about the relevance of learning some of the skills and concepts taught in the experiment books. Amir and Subramaniam (2014) cited an example of their observation of students asking questions on how learning the skills in using the vernier caliper to measure the internal and external diameter of a test tube and compact disc (as described in their activity books) would be useful for them in their daily lives. The use of a STEM-PjBL could address some of these concerns in motivating students to learn science (Elkind 1999). However, the choice of the project to be presented to students does matter. Ideas from educators have shown that the use of projects that kindles interest in students does make a difference in motivating them to be interested in doing the projects (Amir 2014; Kangas 2010; Lan 2011; Meyer 2012; Resnick et al. 2000).

A way for science teachers to get students excited in learning science through projects is to infuse play into lessons. ‘Play’ is a critical issue to consider when introducing science concepts as a means to make learning science fun (Stables 1997). Play also promotes innovation and stretches students’ imagination to foster creativity (Parker-Rees 1997). Play factors can be infused into lessons that present both elementary and complex science concepts. An example of a complex physics concept that has the play factor embedded is shown by Sabin et al. (2008). In the example, the authors described how they made use of a context of superheroes belonging to different groups to present complex concepts in quantum physics. Merging elements of ‘play’ to physics, as shown through such a move, managed to generate interest in students to solve problems in physics. Another way to infuse elements of ‘play’ is through the use of toys. Using toys excites students and builds up their enthusiasm to learn science concepts (Güémez et al. 2009; Featonby 2005; McGartland 1998; and Parker-Rees 1997). Toys are also not limited by a certain language and are thus suitable for use across all ages and cultures (Yau and Wong 2004). Books have been published to show how teachers can inject fun into their lessons by teaching science through the use of toys (McCullough and McCullough 2000; Sills 1999; Sarquis et al. 1995; Sarquis et al. 1997; Sumners 1997; Taylor et al. 1995). Examples of how elements of ‘play’ , such as the use of toys, have been incorporated in the crafting of teaching strategies through STEM-PjBL projects have been described in the literature. These toys can be made from low-cost everyday objects and materials (McGervey 1995). For example, Amir and Subramaniam (2009) described how students get excited about learning physics when they were guided to design and make a low cost candy floss kit gadget through the use of Aluminium foil and objects which are commonly available in the kitchen and a typical physics laboratory. Featonby (2010) and Thompson and Mathieson (2001) showed how ‘playful’ hands-on activities can generate students’ understanding in concepts related to optics through making a ‘magic mirror coin box’ toy that can be easily made using simple paper box and a plastic mirror. Oliver and Ng (1999) described how students were made to understand the concept of forces and elasticity by designing and making rubber-band driven toy aeroplanes. Subramaniam and Ning (2004) described how students were made to understand the concept of resonance by making a simple toy that is made with a wooden rod, strings and pendulum bobs. Zubrowski (2002) carried out a study with students using simple materials to make model windmills in getting them understand several science concepts related to forces and motion. Amir and Subramaniam (2006; 2007; 2014) have also described that students were able to not only able to acquire physics content through designing and making toy projects but also in being able to express their creativity through science knowledge in coming up with their own versions of toy designs.

### Theoretical framework and purpose of study

Since 2004, Malaysian universities have started research on project-based teaching and learning approaches in the engineering fields (Khair et al. 2011). However, STEM-PjBL approaches have yet to be developed for schools, indicating research needs about teachers’ understanding and implementation of STEM-PjBL approaches in science teaching. The literature suggests that a STEM-PjBL approach through designing and making science-based toys can be a useful pedagogy to teach science and create students’ excitement and motivation toward learning science. The researchers believe that when teachers are enabled to engage with such an approach, they would develop STEM-PjBL teaching and learning activities that could set a positive STEM culture in schools. On a bigger scale, implementing a STEM-PjBL approach in science teaching could be a catalyst towards achieving the Malaysian transformation agenda in sparking student interest in STEM subjects.

A professional development workshop approach for pre-service and in-service teachers is one way of creating teachers’ awareness of science teaching through a STEM-PjBL approach (Laboy-Rush 2007). Knowledge of these teachers’ views on STEM-PjBL approaches could inform innovative ways of teaching STEM subjects.

The purpose of this study therefore was to investigate the perceptions of Malaysian science teachers in adopting a STEM-PjBL approach in teaching science. A professional development workshop would provide the platform to engage science teachers in STEM-PjBL approaches to science teaching through designing and making science-based toys.

In formulating research questions, the researchers focused on:

-  Examining teachers’ initial perceptions of a STEM-PjBL approach in teaching science,
-  Exploring their responses to the professional development experience in using the STEM-PjBL approach (namely the benefits and challenges they faced in the workshop), and
-  Exploring the factors that would motivate them to implement the STEM-PjBL approach in the teaching of science in their schools.

The research questions guiding this study are:

1. How would teachers’ perceptions of PjBL in STEM approach evolve as a result of their participation in this STEM-PjBL workshop?
2. What benefits are gained by teachers through engaging with the workshop?
3. What challenges do teachers face as they engage with the workshop?
4. What challenges would teachers potentially face in implementing a STEM-PjBL approach in their classrooms? What suggestions would they offer to overcome these challenges?
5. What would motivate teachers in applying a STEM-PjBL approach in teaching science in their own classes?

## Methodology

### Research design

A two-day professional development workshop was carried out to expose teachers to a STEM-PjBL approach in teaching science. A mixed method approach used qualitative and quantitative methods to address research questions and how teachers could put into practice what they learned in the workshop.

### Time frame and sample

Two weekend days were allocated for workshop. The eight-hour workshop design was the same each day. On the first day (Saturday), 25 pre-service science teachers were involved. They comprised five males and 20 females aged between 20 and 23, and had no prior teaching experience in schools. On the second day (Sunday), 21 in-service teachers were involved. They comprised six males and 15 females aged between 32 and 40, and have 8 to 14 years of teaching experience in primary schools. Eighty-one per cent of in-service science teachers had no former experience with PjBL, whereas 19% sometimes incorporated project-based teaching approaches in their science lessons.

### Collaboration with the workshop facilitator

The facilitator was a Senior Teacher from Singapore with a doctorate in science education, who has adopted a STEM-PjBL teaching approach with his science and Design & Technology (D&T) students for over nine years. Collaboration between the university and the facilitator started six months prior to the workshop. The agreed main approach was to take participants through the classroom teaching practices he adopts which mainly involves students learning science through designing and making toys. The necessary workshop materials were then made ready.

### Data collection

Research instruments included survey forms (quantitative data) and interviews (qualitative data). This approach aimed to provide a holistic view on tracking participants’ perceptions of and reaction to the STEM-PjBL approach.

The survey forms used a six-point Likert scale; Strongly Agree (SA), Agree (A), Slightly Agree (SLA), Slightly Disagree (SLD), D (Disagree), to SD (Strongly Disagree). Quantitative data were collected using pre-workshop and post-workshop surveys. The pre-survey aimed to capture participants’ prior experiences, comfort level and initial perceptions about PjBL in a STEM approach in their instruction. The post-survey aimed to capture participants’ workshop experiences of the STEM-PjBL approach. Survey items were developed from ideas gathered from the literature such as instruments used in studies by Cook and Buck (2013), Ingram and Nelson (2006), Mujtaba and Reiss (2014), Teo (2008), Zwiep and Benken (2013). An open-ended question “What is your overall impression of the workshop?” was stated in the post-survey form in view of obtaining written feedback on the participants through the workshop. The instruments were reviewed by a Science lecturer and Science teacher. Since English was not the common language used by the participants, the researchers translated the instruments into Malay; these were reviewed by a Malay language teacher.

Qualitative data were gathered through (a) individual interviews (20–30 minutes), (b) workshop feedback (open question in post survey), and (c) open discussions at the end of the workshop (60 minutes). The qualitative tools sought participants’ ideas about the benefits and potential challenges they might face carrying out a STEM-PjBL approach and the likely recommendation of STEM-PjBL approach to other teachers in secondary and primary school classrooms. Structured interview protocols were employed with further probing questions following each interview question. Interviews were audio taped and transcribed.

Table 1 shows the tools that are being used to address the corresponding research questions. A group of 15 undergraduate science students were asked to comment on the readability of the items in the data capturing tools. The students agreed that all items were relevant and they should remain in the study.

**Table 1:** **Tools being used to address the research questions**

Research question	Data capturing tools
1	i. Pre-post survey
	ii. Interview question administered at the end of the workshop:
	‘Reflecting on your own experience as a learner and as a teacher, what is your overall reaction to the approach shared in this workshop as compared to existing instructional methods?’
	iii. Open question in Post survey:
	‘What is your overall impression of the workshop?’
2	i. Post survey
	ii. Interview question administered at the end of the workshop:
	‘What particular benefits do you get while engaging in this workshop?’
	iii. Open question in Post survey:-
	‘What is your overall impression of the workshop?’
3	i. Interview question administered at the end of the workshop, and
	ii. Open discussion in class
	‘What particular challenges do you face while engaging in this workshop?’
4	i. Interview question administered at the end of the workshop, and
	ii. Open discussion in class
	‘In your opinion, what kind of challenges will you potentially face in implementing a STEM-PjBL approach in classroom? Please give your suggestion in order to overcome these challenges’.
5	i. Post survey, and
	ii. Interview question administered at the end of the workshop
	‘Would you like to recommend other teachers to apply a STEM-PjBL approach in teaching science in their own classes? Please give your reasons why you like to do so’.

### Method of analysis

For quantitative data, the difference in the percentage score between the pre-survey and post-survey was computed as a measure of change in participants’ perceptions. The index of reliability of the pre-survey and post-survey was 0.96 and 0.92 respectively.

For written and verbal qualitative data, the researchers used interpretive methods (Erickson 1986) to explore common themes that emerged out of 46 participants’ statements and words. An iterative process of coding, memo writing, focused coding, and integrative memo writing (Emerson et al. 1995) was followed. In the Findings’ quotes in italics are from workshop participants.

### Procedures

The workshop facilitator guided participants on how science could be taught and learned through five science-based toy projects discussed in the literature. Examples included teaching concepts of:

-  *Forces*; through a toy balloon helicopter, as described by Ng et al. (2002),
-  *Resonance*; through simple materials (Subramaniam and Ning 2004), and
-  *Thermal physics and chemistry*; through a Candy Floss Kit (Amir and Subramaniam 2009).

In a toy solar car project, participants were taken through a series of hands-on experiments to offer ideas for presenting science content to their own students. The science content for the toy solar car project included:

-  *Mechanism Concepts* - Pulleys, Friction, Speed (including skills in using a stopwatch) and aerodynamics.
-  *Electrical Concepts* – Circuit assembly, circuit drawings and diagrams, and energy conversions.
-  *Structural Concepts* – How layout of components affects weight distribution and car performance.
-  *Concepts in Environmental Awareness and Clean Energy* - Air pollution and ways to reduce it through the use of catalytic converters fitted to cars, and promoting recycling.

The participants were also exposed to creative thinking techniques to support delivery of science content. For example, in the Cartesian diver toy project, participants were guided through ways of teaching the concept of density, while also developing variations of the toy through physics principles, similar to the strategy described by Amir and Subramaniam (2007; 2014). Interspersed with these activities, the facilitator showed a variety of multi-media clips to link concepts relevance to the real world.

Participants were also exposed to design portfolios as a way to record the learning of science as they went through the projects. They were shown how this technique could help students who are more visual-spatial than visual-linguistic oriented (Amir 2014; Amir and Subramaniam 2012; 2014). At the end of the workshop, participants had five toys and two completed portfolios to bring home.

### Findings

#### Quantitative analysis

Pre-workshop survey findings showed that only 15% of participants had some ideas about STEM, while 85% had never heard about STEM approaches to teaching science. The pre-survey also revealed that only seven pre-service teachers perceived STEM as an integrated approach which showed connectedness in the teaching and learning of science, technology, engineering and mathematics that promote higher-order thinking.

*“STEM is a combination of four subjects, namely science, technology, engineering and mathematics, and they are integrated into the teaching and learning process in schools. STEM approach to train higher-order thinking students (HOTS)”.*

Four pre-service teachers were aware of the potential of such an approach for developing creativity and critical thinking among students, namely through participating in active ‘hands-on’ activities.

*“STEM approach in teaching science is an approach that requires students to be more active, particularly in ‘hands-on’ activities through project-based learning. This approach also requires students to think critically and creatively”*.

#### Findings for research question 1

How would teachers’ perceptions of PjBL in STEM approach evolve as a result of their participation in this workshop?

#### Participants’ initial perception of STEM PjBL approach prior to the workshop

Pre-survey responses (Table 2) indicated that:

**Table 2:** **Summary of percentage, means and standard deviations for response of pre-service and in-service teacher in pre-survey**

			Strongly agree	Agree	Slightly agree	Slightly disagree	Disagree	Strongly disagree	M	SD
1.	I believe that design is an important element when teaching topics related to STEM subjects.	P	32	**68**	-	-	-	-	5.32	.48
		I	**62**	33	5	-	-	-	5.57	.59
2.	I incorporate a project-based teaching approach in some of my science lessons.	P	16	**68**	16	-	-	-	5.04	.61
		I	19	**57**	19	5	-	-	4.90	.77
3.	I am comfortable using a project-based teaching approach in my science lessons.	P	20	**48**	32	-	-	-	4.92	.76
		I	14	**62**	19	5	-	-	4.86	.73
4.	I believe that my students learn science better through a project-based teaching approach, as compared to frontal teaching.	P	**52**	36	8	4	-	-	5.36	.81
		I	**71**	24	5	-	-	-	5.62	.59
5.	I believe that all science topics that I have to teach can be taught through a project-based teaching approach.	P	16	**40**	36	8	-	-	4.64	.86
		I	4	**48**	33	-	5	-	4.62	.86
6.	I believe that a project-based teaching approach in science lessons can help my students to think creatively.	P	**64**	36	-	-	-	-	5.64	.49
		I	**71**	24	5	-	-	-	5.62	.59
7.	I believe that my students will be interested in learning science through a project-based teaching approach.	P	48	**52**	-	-	-	-	5.48	.51
		I	**71**	24	5	-	-	-	5.62	.59
8.	I believe that students will be motivated to learn science when they can see a link of learning science to other subjects.	P	44	**56**	-	-	-	-	5.44	.51
		I	**67**	33	-	-	-	-	5.62	.50

*’P’ represents Pre-service Teacher; ‘I’ represents In-service Teacher.

-  100% teachers either strongly agreed or agreed with the statements, *“I believe that students will be motivated to learn science when they can see a link of learning science to other subjects”.*
-  100% pre-service and 95% in-service teachers either strongly agreed or agreed with the statements, *“I believe that design is an important element when teaching topics related to STEM subjects”, “I believe that a project-based teaching approach in science lessons can help my students to think creatively”*, and *“I believe that my students will be interested in learning science through a project-based teaching approach”*.
-  84% pre-service and 76% in-service teachers either strongly agreed or agreed with the statement, *“I incorporate a project-based teaching approach in some of my science lessons”*.
-  68% pre-service and 76% in-service teachers either strongly agreed or agreed that they were comfortable using a project-based teaching approach in their science lessons.
-  88% pre-service and 96% in-service teachers either strongly agreed or agreed that, *“I believe that my students learn science better through a project-based teaching approach, as compared to frontal teaching”*.
-  56% pre-service and 52% in-service teachers either strongly agreed or agreed that, *“I believe that all science topics that I have to teach can be taught through a project-based teaching approach”*.

Pre-survey analysis found that pre-service teachers’ perceptions differ slightly from in-service teachers about STEM-PjBL approach. Pre-service teachers’ survey responses were based on their knowledge from their training, theory, and past experiences, without solid STEM-PjBL experience in the classroom. For in-service teachers, with more classroom experience, there was a lower consensus than pre-service teachers on using a STEM-PjBL approach in science lessons. In addition, a slightly lower percentage (52%-56%) of in-service and pre-service teachers agreed with the view that all science topics can be taught through a project-based teaching approach.

#### Participants’ perception of STEM PjBL approach after workshop

Post-survey responses (Table 3) indicated that:

**Table 3:** **Summary of percentage, means and standard deviations for response of pre-service and in-service teacher in post-survey**

			Strongly agree	Agree	Slightly agree	Slightly disagree	Disagree	Strongly disagree	M	SD
1.	This workshop provided me with ideas to present science content in ways that will appeal to my students.	P	**76**	24	-	-	-	-	5.72	.46
		I	**95**	5	-	-	-	-	6.00	.00
2.	This workshop provided me with ideas to present science content in ways that match the learning styles of my students.	P	**64**	28	8	-	-	-	5.52	.65
		I	**81**	19	-	-	-	-	5.76	.44
3.	This workshop provided me with ideas on how to foster creativity amongst my students.	P	**76**	24	-	-	-	-	5.76	.44
		I	**95**	5	-	-	-	-	6.00	.00
4.	I believe that the approach shared in this workshop will make my students motivated in learning science.	P	**67**	33	-	-	-	-	5.68	.48
		I	**95**	5	-	-	-	-	5.95	.22
5.	I find this approach easy to implement in my lessons.	P	**36**	36	28	-	-	-	5.12	.78
		I	38	**62**	-	-	-	-	5.33	.48
6.	I am confident on carrying out the projects in my science lessons.	P	28	**60**	12	-	-	-	5.20	.58
		I	43	**57**	-	-	-	-	5.42	.51
7.	I find this workshop useful in providing teaching strategies to teach science to my students.	P	**44**	48	8	-	-	-	5.36	.64
		I	**71**	29	-	-	-	-	5.71	.46
8.	I will recommend other teachers to learn about teaching physics content through designing and making science-based toys.	P	**56**	44	-	-	-	-	5.60	.50
		I	**82**	18	-	-	-	-	5.81	.40

*’P’ represents Pre-service Teacher; ‘I’ represents In-service Teacher.

-  100% teachers either strongly agreed or agreed with the statements, “*The workshop provided me with ideas to present science content in ways that will appeal to my students*”, “*The workshop provided me with ideas on how to foster creativity amongst my students*”, “*The approach shared in this workshop will make my students motivated in learning science*”, and “*I will recommend other teachers to learn about teaching science content through designing and making science-based toys*”.
-  100% in-service and 92% pre-service teachers either strongly agreed or agreed with the statements, “*The workshop provided me with ideas to present science content in ways that match the learning styles of my students*”, and “*I find this workshop useful in providing teaching strategies to teach science to my students*”.
-  100% in-service and 76% pre-service teachers either strongly agreed or agreed with the statement, “*I find this approach easy to implement in my lessons*”.
-  100% in-service and 88% pre-service teachers either strongly agreed or agreed that they were confident in carrying out the projects in their science lessons.

The post-survey findings show a substantial increase in the ‘Strongly Agree’ category and the mean scores after the teachers participated in the workshop. Compared to pre-service teachers, in-service teachers in fact had greater agreement on the ease of implementing STEM-PjBL in their lesson and were slightly more confident in carrying out the projects in their science lessons.

### Qualitative analysis on participants’ response

#### Participants’ reflection on STEM-PjBL approach

Participants’ extended views about STEM-PjBL approach were also gathered from interviews and open questions in post survey to support survey responses. Several themes emerged as follows:

#### A fun, interesting, enjoyable and exciting approach

Twenty eight participants felt that learning science via designing and making science-based toys had offered a learning environment that was fun, interesting, active, enjoyable and exciting. Participants expressed the view that the STEM-PjBL approach was focused and not boring. A sample response from interview was included as below:

-  *“…project-based toys making is very interesting and could foster students to be more active and interested in learning science”* (PT20; IT13).
-  *“The approach used in workshop is: more fun, amused, and attractive than the one we used in classroom”* (PT01; PT04; PT25; IT01; IT02); *“more interesting, focused and not boring”* (PT05; PT11; PT14; IT09; IT11); *“more enjoyable, interesting and easy to understand”* (PT06; PT17; IT06); *“is fresh and exciting… opened our mind to lead the students to understand an abstract concept”* (PT03; PT13).

#### Offers opportunities to be creative

Participants noted that learning with science-based toys is not merely playing with a toy to have fun. They believed that designing and making their own science toy is a promising approach that offers opportunities for students to create something novel and develop creative thinking skills. Sample responses include:

-  *“Students have opportunity to explore their own creativity to create something new. Creative ideas are derived from hands-on activities in designing and making a toy”* (PT02; PT16; IT02; IT09).
-  *“The shared approach was promising and encouraging students to think creatively about Science”* (PT06; PT11; IT17; IT21).

Similarly, participants learned how to teach creatively, stating that when applying such an approach, it did help teachers to consider creative ways of conveying science content.

-  *“Increase our knowledge how to be innovative and creative in teaching science and not just focus on theory …”* (PT03; IT16).
-  *“The approach provides creative ideas for T&L as well as enhancing creativity among students”* (PT25; IT13).
-  *“I learned to design and make the model and toys for science lessons. …to be more creative and able to cultivate a passion for improving student performance”* (PT17).
-  *“…increased my knowledge on how to use recycled materials creatively in producing learning aids”* (PT15; IT10).

#### Attract students’ interest and attention

The workshop PjBL activities enhanced students’ interest as reported by eighteen participants:

-  *“This approach is able to attract and increase students’ interest in learning Science”* (PT02; PT03; PT06; PT07; PT10; PT12; PT20; IT06; IT07; IT10; IT15); *“…It can bring up high commitment to learn and increases interest in learning”* (PT13; IT11).
-  *“…. able to attract interest and make students understand the concepts”* (PT14; IT20); *“…. and will eventually increase their understanding and improve their grade in examination”* (PT01; IT17); “*…foster interest among students who have problem in understanding the Science subject”; ‘This can attract students to learn something new and can be applied in their everyday life*” (PT22).

Participants also remarked that the approach helped guide the teacher on ways to attract students’ attention, especially those who have less interest in learning Science. In their words:

-  *“A good approach to guide teacher how to attract students’ attention in learning”* (PT07); *“can attract students who are not interested in learning science and understand the concept easily”* (PT15; IT5); *“will definitely attract attention of students and increase their desire to learn, and further strengthen their knowledge”* (PT23).
-  *“Learning that involves’toys’ attract students and make students more focused in their learning”* (IT21).

#### Increased motivation to learn

After one day of involvement with the STEM-PjBL workshop, participants believed that STEM-PjBL is able to increase motivation of students to learn science and also motivate teachers to strengthen and improve their teaching and learning:

-  *“STEM-PjBL encourages students to learn more actively and increase motivation to learn”* (PT02; IT21).
-  *“The approach is suitable and able to motivate students to learn”* (PT11; IT03).
-  *“More relaxed approach, to motivate students and to expose students to inquiry findings”* (IT02).
-  *“… can motivate and inspire teachers to strengthen and expand … teaching and learning”* (IT12).

Students gain motivation as well when they are stimulated by their peers who develop self-esteem, as suggested in participants’ interview below, is critical:

-  *“Through this approach, each student will have their own opportunity to create a solar car (and) encouraged to place their toy car to move on the floor. If the student failed to perform a proper product, they will feel … disappointed (and) therefore, motivated to try hard to complete the activity”* (PT16); *They will be motivated as if they saw effort of their peer to produce a workable product.”* (IT19).

#### Developing problem-solving skills

Three participants expressed a view that the workshop encouraged them to find their own solutions for designing and making projects. They realized the STEM-PjBL provides students with opportunities to work on their own with instructor guidance:

-  “*I noticed students are given the opportunity to find their own solutions in the activities of making a solar car, with the guidance from teachers*” (PT01; PT10).
-  “*Students have to solve a given problem using their own knowledge/ideas”* (PT02).

The participants learned that a STEM-PjBL approach enables teachers to act as a facilitator and provide students with a process to retrieve knowledge and generate solutions, in ways that support and develop their problem- solving skills.

#### Supports learning about environmental values

The hands-on workshop activity proved to be a catalyst for thinking about how to develop easily accessible, inexpensive material to create a meaningful project based learning. Participants’ noted that:

-  *“*…*the materials used are simple and inexpensive, but it can produce interesting and meaningful lessons”* (PT24; IT21*)*; *“assists in the preparation of the material in order to carry out hands-on activities in the classroom”* (IT20*); “can be applied in teaching science”* (IT13).

Some comments especially referred to material that can be recycled and how STEM-PjBL not only helps participants to understand science, but also supports learning about environmental values such as recycling; *“… material used in the project based toy are easily available, and can promote a sense of recycling among the students”* (PT01; PT18; IT04).

#### Positive applicability and suitability of STEM-PjBL in learning science

It is worth mentioning that all participants felt positive and encouraged by the STEM-PjBL approach with a strong determination to apply it in their future teaching. Sample responses include:

-  “*I hope this approach will be continually conducted in future classroom because it is a very effective in motivating the learners in a creative learning environment”* (IT06); *“An exciting approach suitable to be used …in schools”* (PT15); *“Many of the skills and methods learned in the workshop are applicable in teaching and learning in school”* (PT11).
-  *“I can relate each undertaken activity to science and I want to teach using this method in future”* (PT10; PT24); *“definitely something new and useful … to use in school”* (PT21; IT05).

Four participants wished to extend the STEM-PjBL workshop to other teachers throughout urban or rural areas; *“This workshop has given me a lot of input about effective methods of teaching science. I hope this workshop can be extended and introduce to all science teachers in urban and rural schools”* (IT04; IT06; IT12; IT16).

In addition, they also claimed that STEM-PjBL approach is very useful in putting the latest Standard Curriculum for Primary Schools (KSSR) into practice. As one participant remarked: *“I hope that this workshop can also be used for Mathematics. Workshops like this are very useful to the practice of new curriculum of the KSSR”* (IT02).

In sum, all 46 science teachers involved in the workshop had a positive regard for the applicability of a STEM-PjBL approach, and their perceptions were positively changed after the workshop. Their post-survey reflections and feedback during interviews pointed out that the workshop STEM-PjBL activity brought considerable fun, joy, and excitement. It could attract students’ attention and interest, and enhance motivation and creativity in science learning. In essence, a STEM-PjBL approach seems to support teachers in delivering science content in a more appealing way, promote positive environmental values and develop problem solving skills.

#### Findings for research question 2

What benefits are gained by teachers through engaging with the workshop?

#### Opening up one’s mind to designing and making science-based toys

The workshop illustrated the usage of science-based toys such as solar car in the sciences classroom. Participants considered that the learning science by designing and making science-based toys opened their mind about science teaching:

-  *“It has opened our mind how to guide students to understand an abstract concept”* (PT03; PT13); *“to teach weak students using an effective way”* (PT03); *in creating strategies for more effective science teaching,*” (PT11); *to explore a wider view in teaching Science, especially in handling a science project*” (IT16);*“to have an idea of project based learning strategies”.*

Participants’ feedback during interviews suggests that the workshop offered them new ideas and skills about design and its application in science classes:

-  *“Gained new experience, knowledge and skills of design in carrying out project activities for science teaching and learning”* (PT06; PT12; PT15; IT14).
-  *“The teaching methods and techniques using hands-on activities increase my knowledge about the importance of design and how to apply them in teaching”* (PT01).

The comments reveal participants’ open-mindedness to new teaching approaches that apply designing and making science-based toys in science classroom.

#### Acquired new experience for making science classrooms more effective

Participants felt that the workshop offered them new ideas and experience to an effective Science classroom. Below are some of their comments:

-  *“The workshop was very helpful, especially for me who still does not have teaching experience. I learned ideas to present science content so that students are more interested in science”* (PT02).
-  *“… I got a lot of benefits that can be applied while teaching students …provides interesting ideas and stimulates my thinking to deliver more effective teaching”* (PT07).
-  *“This workshop provided a lot of interesting and creative science activities. Hope this workshop can be implemented in the future as it helped provide ideas to the teachers to make teaching and learning process in interesting and effective ways”* (PT09; PT14; IT13; IT21).

Additionally, participants felt that they had learned to use design portfolios to summarise students’ learning outcomes, noting the simplicity of grasping and reviewing concepts with the portfolio:

-  *“I learned the method of using the portfolio that can make students better understand what they are learning”* (IT01); *“The portfolio is also a neat way to summarize and organize the students’ learning outcome which the students can refer back to, in contrast to the complex explanation (in students’ perspective) in physics textbook”* (IT04, IT11).

Two participants (IT12; PT16) said that they acquired knowledge in diversifying science activities in school, including strategies to prepare a more attractive lesson:

Some teachers were aware of the potential of STEM-PjBL approach for acquiring new knowledge through participating in “hands-on” activities centered on building artefacts:

-  *“Students learn actively through the process of designing and building of artefacts which made them to come out with their own product”* (PT08).
-  *“I am able to construct new knowledge through hands-on activities”* (PT01; PT03; PT06; PT08; PT09; IT07).
-  *“Students experience themselves the processes of constructing new knowledge, for example, building a solar car starting from assembling the wheel to the building of a car frame”* (PT12).

#### Fostering creativity and thinking skills

Four teachers noted that a systematic project can help to enhance their students’ thinking skills, demonstrate the advantages of problem-based collaborative projects for weaker students:

-  *“Using a systematic project can help learners to improve their thinking skill. We were able to increase our knowledge in using problem solving collaborative project as teaching methods to weak pupils”* (PT10; PT15; IT04; IT16).

Participants’ feedback indicated that a STEM-PjBL approach could engage students to showcase their creativity when students design and improve their products. It also drives teachers and students to thinking critically and creatively in making use of recycle materials for science activities.

-  *“The student’s creativity can be … cultivated through project based learning when students use their own ideas to design their projects and to improve the quality of their products. For example, students design their own “solar car” so that the car is able to function more effectively”* (IT19).
-  *“Even though teachers could be facing budget problems, however, it drives teachers and students to use their critical and creative thinking skills in making use of recycle materials”* (IT08).

Most of the pre-service teachers felt that the brainstorming session used in the STEM-PjBL approach helped them gain methods and skills to foster student participation in generating ideas, particularly among weak students:

#### Inspired to teach in an innovative way

The STEM PjBL workshop provides an impetus for participants to consider innovative teaching practice and felt motivated to extend the workshop and facilitator’s ideas in their own teaching. Their responses included:

-  “*The workshop was very good to motivate prospective teachers in using an innovative way of teaching and learning”* (PT01; PT17).
-  *“Through this workshop I was able to learn new ways of teaching. Such techniques are very rarely applied in any school that I’ve ever seen. From here, the speakers gave me the motivation to become an innovative teacher”* (IT11; IT19).
-  *“Activities such as these should be conducted as often as possible and involve teachers in all areas. Teachers from different areas or fields must also be involved. Promoting of these activities can motivate and inspired the teachers to strengthen and expand the idea of teaching and learning”* (IT16).

#### Recognizing the interdisciplinary nature of STEM-PjBL approach

Participants mentioned the interdisciplinary nature of STEM-PjBL approach and the value of project work in engaging students. The STEM-PjBL approach allows teachers to integrate several related subjects into one specific scientific project. One in-service teacher said; *“A good point of this approach is that it can cover several subjects simultaneously within one project”* (IT14). Other teachers also recognized that STEM-PjBL allows learning happen across different disciplines:

-  *“This workshop involved learning across curriculum as well. As an example, knowledge of physics, science and technology are needed during the constructing of solar car”* (IT12; IT13).
-  *“STEM-PjBL approach is able to test and improve skill of student because it involves various topic and subject”* (IT15; PT03); *“to include many topics from different subjects, such as science, physics and mathematics”* (IT17; PT13; PT19).

Other pre-service teachers’ responses in open discussion included:

-  *“The workshop will also involve learning across the curriculum. Example: during the construction of a solar car, knowledge in other subjects such as mathematics, science, and technology are needed”* (PT06; PT09).
-  *“The STEM-PjBL approach is also able to test and improve student’s skills because it covers various topics and subjects from science, physics and math”* (PT07; PT08).

The workshop also demonstrated the interdisciplinary nature of STEM as teachers learned to bridge divisions among different learning areas through a single project. The relevance of PjBL helps participantsto see a link between different learning areas as one participant noted: *“I especially like the way we can use a single project to cover wide learning areas in physics because of its relevance and simplicity in approaching students who are struggling to understand physics concepts cognitively through textbook”* (PT12).

In sum, the workshop demonstrated the advantages of the STEM-PjBL approach to teaching for science teachers. Participating teachers gained new ideas and skills applicable to an effective science classroom, such as designing and making science-based toys, fostering their own and their student’s creativity and thinking skills, and teaching in innovative ways. The workshop demonstrated the interdisciplinary nature of a STEM-PjBL approach and the benefit of design portfolios, brainstorming, and diversifying activities in teaching science.

#### Findings for research question 3

What challenges do teachers face as they engage with the workshop?

Participating teachers noted several challenges while carrying out workshop activities. In particular, they mentioned that implementing PjBL activities was not as easy as they first thought and that it required systematic planning.

#### Insufficient time to complete tasks

Forty three percent of participants said that they had insufficient time to complete the suggested hands-on activity and that things seemed chaotic for them if the workload increased alongside time constraints. Sample responses include: *“…I feel chaotic due to insufficient time for two activities that are performed concurrently”* (PT01; PT12; PT16; PT17: IT04; IT09).

Other comments included:

-  *“Facilitator provided clear information, but time is very short. Students are only able to do the experiment in a short time and in hurry”* (IT03).
-  *“…Facilitator are very clear in describing the scope of the program and procedures. Tools are available and the learning is enjoyable. However, it takes a relatively long time for a single project”* (IT16).

As a consequence, they suggested the workshop be extended to a longer period for more hands-on activities to be demonstrated and accomplished.

-  *“Execution time for this workshop should be extended so that participants have sufficient time to complete the activity. More activities should also be provided to help increase teachers’ knowledge*…*”* (IT15).
-  *“It is better to have more time doing the project based learning. If we have enough time, I think we can do all the activities without rush”* (PT01; PT12; IT17).

#### Language as a communication barrier

The program was conducted mainly in English. However, Malay is the main language of instruction in Malaysian national school classrooms. Participants said that they felt uncomfortable raising questions in English: *“There are so many questions I wanted to ask but I was not able to raise because of the [language] barriers that I had”* (PT24; IT01; IT05; IT14). There were also some difficulties in understanding the facilitator’s instructions: *“We are not clear with the explanation given”* (PT23; IT14; IT16).

#### Unexpected conditions that contributed to unsuccessful outcomes

The day that project based activities were carried out, several unexpected situations affected progress. Specifically, clouds prevented some participants demonstrating the solar car activity and they noted that *“… weather conditions can also affect us when doing experiments”* (PT03; PT07; IT06; IT13; IT20). In addition, three participants pointed out that the outcomes of activities were less exciting as they did not meet expectations. Two participants persevered repeating the activity several times to achieve the desired outcome.

#### Lack of subject matter knowledge in a STEM-related field

Participants held different teaching options including Science, Physics, Biology, Chemistry and Mathematics. The facilitator applied mostly Physics concepts for the activities leading to some difficulties among participants who lack Physics familiarity.

-  *“Difficult to recall laws/theory to be applied during the workshops”* (IT03; IT 06; IT13; IT16; IT19; IT 20).
-  *“Teachers who have no basis in Physics will have some trouble in understanding the concepts”* (IT01; IT07; IT08; IT14; IT 17; IT18).

In addition, 80% of the participants addressed the importance of acquiring knowledge in engineering and technology in order to successfully carry out activities.

In sum, the challenges facing the participants while carrying out workshop activities included workshop time constraints, language barrier in communication, unexpected external conditions and lack knowledge of subject matter in a STEM-related field.

#### Findings for research question 4

What challenges would teachers potentially face in implementing a STEM-PjBL approach in their classrooms? What suggestions would they offer to overcome these challenges?

Following the experience gained in the complete session of the professional development workshop, most participants expressed their worries on the following challenges they would potentially face in implementing a STEM-PjBL approach in their classrooms as explained as follows:

#### Inadequate materials and facilities

Ninety six percent of the participants noted their challenge to obtain science-based toys, a solar car for example, since it is rare for solar panels to be sold as single items. Participants serving in rural schools reportedly face more serious problems compared to urban contexts. In addition to the difficulty in obtaining experimental materials, 50% of the participants mentioned that school laboratories in rural areas lack maintenance and equipment provision. Because of this, participants proposed using readily available recycled materials to make science-based toys or to use material that is easier to find*.*

Seventeen participants reflected on the amount of material needed when activities are carried out in a large class. In general, an ordinary classroom in the urban Malaysian schools has around 40 to 50 students. Thus, teachers have to look for a large amount of science-based toy materials to cope with the needs. Participants suggested conducting activities in smaller groups to reduce the quantity of materials needed.

Ninety one percent of the participants suggested that students and school stake holders could take their own initiatives to obtain relevant materials. They stated, *“The students can be asked to bring materials themselves. School or teacher should attempt to provide material or prepare budget for the purchase of equipment or collaborate with other agencies/parties for the needed material”*.

One in-service teacher proposed that the Malaysia Education Department should develop a basic project design which could incorporate the entire syllabus in one project.

#### Classroom time constraints

One in-service teacher stressed the importance of allocating time in favor of project-based learning. In fact 87% of the participants felt that a relatively longer time should be allocated to carry out the hands-on activities that utilized STEM-PjBL approaches because:

-  Time allocated for science lessons is too short;
-  Students need more time to understand the instructions;
-  Special materials are needed;
-  *STEM-PjBL involves a wide range of cognitive levels*.

One participant believed that students would require more time beforehand to understand the instruction on the execution of the experiment. Undoubtedly, adopting STEM-PjBL approach in a science classroom requires appropriate time to carry out the activity. However, the 60 minutes per week classroom time that is set by Malaysian Education Department for science lessons is actually inadequate for such specific activities to be implemented successfully. The low performing elementary schools, in particular, often need extra time for students in comprehending instructions, thus do not provide enough time to adopt an effective STEM-PjBL approach to learning science.

In regard to this, participants recommended to increase the allocation time in science lessons. They suggested that teachers can take initiative to carry out such activities after school hours. Otherwise, school stakeholders could contribute to solutions with extra allocation of time for implementing STEM-PjBL projects.

#### Lack of expertise/knowledge in STEM-PjBL related projects

Sixty five percent of the participants expressed some anxiety about their lack of expertise to conduct a STEM-PjBL activity. Feedback included:

-  *“I Lack experience and knowledge in engineering and design”* (PT09; PT16; IT10; IT15).
-  *“Students may have more knowledge and experience than the teacher when science and technology is put in play”* (IT11; IT13).
-  *“Lack of expertise to teach the way a scientist does”* (PT19; IT13; IT21).

The feedback speaks to some participants’ lack of confidence and need to increase their STEM-PjBL experience. Participants felt that teachers should be exposed to a variety of activities that involve STEM-PjBL related experiment/projects.

Seventy six percent of the in-service teachers’ concerns, in contrast with pre-service science teachers, related more to an anxiety about the ability of primary students to manage activities that require experience or higher order thinking. They noted pupils who are mostly less experienced/knowledgeable in Science may feel hard in carrying out experiments*.* Some believed that the activities demonstrated at the workshop are incompatible with younger students, that they were hard to apply, and probably could not achieve an end product. Therefore, for this group of young students, participants advised giving a prior overview or step-by-step explanation in how to carry out the STEM-PjBL activities.

Additionally, some participants proposed approaching their students one at a time by providing appropriate guidance to overcome this matter.

#### Teaching preparation for less interested students

Before conducting a STEM-PjBL class, teachers need to have well-planned preparation, especially for students who are less interested in science. Seventeen percent of participants note: *“The challenge is to provide a description for the high level activities of STEM-PjBL, especially for students who are less interested in science”.* Thus, participants suggested planning in advance the activities, materials and apparatus that are needed.

#### Cost constraints

Ninety three percent of participants’ feedback indicated that existing financial budgets could not support classroom implementation STEM-PjBL. Forty three percent of participants stressed that Ministry of Education should provide adequate financial support for promoting such an approach, whereas fifty percent suggested doing group activities and hence reducing the amount of materials needed.

The challenges participants described above could hinder the implementation of interdisciplinary STEM-PjBL and highlight the importance of preparation in STEM-PjBL projects to ensure that learning outcomes are met. The participants were keen to adopt STEM-PjBL approaches and suggested further training needs follow this workshop which provided them special exposure and experience.

#### Findings for research question 5

What would motivate teachers in applying a STEM-PjBL approach in teaching science in their own classes?

Participants gave four key drivers motivating them to apply a STEM-PjBL approach to science teaching in their classes - the practical ‘learning by doing’ approach; fostering students’ multiple intelligences; enhanced understanding of science content; and exposure to real life problems.

When participants were asked about their determination to implement STEM-PjBL in their future teaching, all but two showed an urgency to recommend other teachers to learn about teaching science content through designing and making science-based toys:

-  *“It is wonderful to have this experience and I am sure I will bring this idea into my teaching and learning process in school”* (42% of participants).
-  *“Certainly we will recommend other teachers to learn about this approach because it helps to open our mind from practicing traditional teaching method”* (54% of participants).

It seems that participants were able to shed light upon the pros and cons of implementing STEM-PjBL approach in teaching science. The reasons for them to make this move in future science classroom were described as follows:

#### Learning by doing

Students are encouraged to solve problem mainly on their own. *“Student wants to know about the project so they carry out the given project on their own”* (PT07), a teacher said. Recalling the program, a teacher remarked that, *“Students are given opportunity to produce something by applying the learned science concept. They consider the most appropriate decoration, accessories, and designs for the product based on their own creativity”* (IT10). Another teacher indicated that *“when students carry out hands-on activity, they study the problem on their own, getting assistance from peers and teacher. This reflects the nature of student-centred learning style”* (IT11).

Some other comments included:

-  *“Students are granted chances to find solution for the assigned problem by their own, for example building a solar car from the beginning until the end, but under teachers’ guidance and with some assistance from friend”* (PT04; PT24; PT25); *“It includes a lot of hands-on activities”* (PT01; PT07; PT09; IT11; IT15; IT20)*.*

Students will only seek a teachers’ assistance when some matter blocked progression:

-  *“These are student-centred activities and facilitator taught us how to carry out activities. He continued to assist his students but only to provide guidance”* (IT07).
-  *“Teacher only provides raw idea for the implemented hands-on activity”* (IT13).

#### Able to foster learners’ multiple intelligences

Participants expressed their opinion that STEM-PjBL approach helps foster students’ multiple intelligence:

-  *“…help to foster or cultivate multiple intelligence through hands-on activity, for example, sketching a science concept”* (PT04; PT13; PT21)*; through designing, problem solving and constructing the project, e.g.: building a solar car that possesses great aerodynamics.”* (PT04; IT13).
-  *“…acquiring various learning ability instantaneously”* (PT01; IT12*); “drive students to the maximum learning”* (PT09; IT10)*.*
-  *“…show their talent and skill on the assigned project. For example, used various materials and artistic sense to create a layout of creative and attractive attribute of a solar car, whereas for science concept, they design the outer look with the consideration of the car stability and speed”* (PT15).

Findings indicated participants strongly believe that students can develop the abilities of visual-spatial, logical-mathematics, and bodily kinaesthetic during the progress of the activities.

#### Able to enhance the understanding of science content

From participants’ perspectives, the design element of a STEM-PjBL approach helped to motivate students, and to enhance their understanding of science concepts in long-term memory.

-  *“Learning through design helps to consolidate students’ memory about the science content”* (PT8; IT01).
-  *“Yes, learning through design helps to promote students’ understanding and interest”* (PT07; PT13; IT05; IT12; IT17).

They also noticed that PjBL approach could help to ease students’ learning in science, particularly, for those who are struggling to understand concepts through abstract textbook explanations:

-  *“To me, learning using PjBL approach is easier to understand (the content) than the textbook alone. Students’ interest to study Physics will also increase if a lot of these kinds of activities are used”* (PT15).
-  *“I especially like the way we can use a single project to cover a wide learning area in physics because its relevancy and simplicity in approaching students who are struggling to understand physics concept cognitively through textbook”* (PT16).

Through designing and making Science toys, participants believed that it helped *“Students to learn in a way to understanding the concept, not just memorizing the facts”* (IT03; IT04; PT25). Other reviews from participants are shown below:

-  *“Normally, student learned those concepts in class. But, from this workshop, I can see how student is given chance to develop the concepts from the hands-on activity. For instance, in the activity of making balloon helicopter, student is able to apply the learned Newton’s Third Law, to explain how the balloon acted as an action and the air trapped in the balloon as a reaction”* (PT03).
-  *“In a STEM activity, students” capability to explain and create is boosted. In the solar car activity for example, students are prompted to explain why it is necessary to have a larger back wheel than the front wheel”* (PT06).
-  *“This workshop also showed me that no method is applicable to all types of students. Some students are great in physics but unable to develop their potential due to not being taught in the manner that help booster their understanding of physics”* (PT11).

This approach could also “*make students who are not interested in science to understand science concept easily*” (PT05; PT14; IT02). One participant reflected that “*experimenting is an effective teaching method. Through this relevant approach, students can comprehend better Science*” (IT17). In this study context, participants were given opportunity to design and make their own solar cars by getting assistance from workshop facilitator. Thereby, it also provided students an opportunity to develop a deeper understanding in the related content knowledge as noted by one participant, “*Students learn actively through project construction. This approach encourages them to develop their own project. They would be able to visualize the concept applied by drawing the layout of the project*” (IT16).

#### Exposure to real life problems

Participants noted that the STEM-PjBL approach exposed them to some experiences about real world science. Through this exposure they indicated that their students could practice science and technology into daily problem solving.

-  *“…students can practice science in daily life problems”* (PT01; PT09; PT11; IT10); *“exposed students some experiences in applying the theory practically through constructing a project”* (PT01; PT02; IT10; IT18; IT20).
-  *“It could also improve students’ and teachers’ technology skill in future” (*IT02).

The qualitative results from participants’ responses above are summarized in Table 4.

**Table 4:** **Summary of participants’ qualitative responses**

Research question	Participants’ response		Frequency (N = 46)	Percentage
1	*What were participants’ reflection on STEM-PjBL approach?*			
	A fun, interesting, enjoyable and exciting approach		28	61.9
	Attract students’ interest and attention		23	50.0
	Offers opportunities to be creative		15	33.6
	Positive applicability and suitability of STEM-PjBL in learning Science		12	26.1
	Increased motivation to learn		8	17.4
	Supports learning about environmental values		7	15.2
	Developing problem solving skills		3	6.5
2	*What benefits were gained by teachers through engaging with the workshop?*			
	Acquired new experience for making Science classrooms more effective		19	41.3
	Recognizing the interdisciplinary nature of STEM-PjBL approach		15	32.6
	Opening up one’s mind to designing and making science-based toys		10	21.7
	Fostering creativity and thinking skills		6	13.0
	Inspired to teach in an innovative way		5	10.9
3	*What challenges did teachers face as they engage with the workshop?*			
	Insufficient time to complete tasks		20	43.5
	Lack of subject matter knowledge in a STEM-related field		12	26.1
	Unexpected conditions that contributed to unsuccessful outcomes		10	21.7
	Language as a communication barrier		7	15.2
4	*What challenges [C] would teachers potentially face in implementing a STEM-PjBL approach in their classrooms? What suggestions [S] would they offer to overcome these challenges?*			
	[C] Inadequate materials and facilities			
		Difficulty in obtaining experimental materials	44	95.7
		Rural school laboratories lack equipment provision	23	50.0
	[S]	Using readily available recycled or easier accessed materials	44	95.7
	[C] A large amount of experimental materials to cope with large class		17	36.9
	[S]	Conducting activities in smaller groups	17	36.9
		Students and school stakeholders take their own initiatives to obtain relevant materials.	42	91.3
	[C] Classroom time constraints		40	86.9
	[S]	Carrying out STEM-PjBL lessons after school hours	40	86.9
	[C] Lack of expertise/knowledge in STEM-PjBL related projects		30	65.2
	[S]	Exposing teachers to STEM-PjBL training	30	65.2
	[C] Teaching preparation for less interested students		8	17.4
	[S]	Planning in advance the activities, materials and apparatus	8	17.4
	[C] Cost constraints		43	93.4
	[S]	Doing group activities	23	50.0
		Getting financial support from Ministry of Education	20	43.4
5	*What would motivate teachers in applying a STEM-PjBL approach in teaching science in their own classes?*			
	Able to enhance the understanding of science content		20	43.5
	Learning by doing		14	30.4
	Exposure to real life problems		10	21.7
	Able to foster learners’ multiple intelligences		10	21.7

## Discussion and conclusion

Findings from this study reveal that the professional development workshop has helped science teachers to expand their insights and build positive perceptions on the use of a STEM-PjBL approach to teach science. The teachers found that the workshop has exposed them to new teaching strategies that offer enjoyable hands-on lessons, such as getting students to design and make science-based toys, in the teaching and learning of science. The teachers believe that this approach could lead to students learning science in ways that excite them, which in turn could promote their interest, motivation and attention in learning science.

In recording the learning of science content through a STEM-PjBL approach, teachers are in favour of the idea of getting students to sketch their toy designs and provide annotations and comments alongside their sketches in describing how knowledge and skills from science contribute to their toy designs. Teachers believed that this approach would not only allow students to gain science content but also in providing an avenue for them to express their creative ideas. Furthermore, teachers felt that this approach caters to the various multiple intelligences in students’ learning as described by Howard Gardner (1983), namely in tapping on the students’ visual-spatial, logical-mathematics, and bodily-kinaesthetic and not just visual-linguistics modes. This could help students who may not usually motivated to learn science through the books to be more interested in learning science. Moreover, as teachers go through the STEM-PjBL approach in the workshop, they felt motivated to learn science themselves, which led to a considerable number of them indicating that they will recommend this approach to other science teachers.

There is no clear evidence in this study to measure how a teacher may improve his or her own creative teaching practices. Nevertheless, 46% of the teachers indicated that the creative learning environment setting in a STEM-PjBL approach could provide a way for science teachers to sharpen their critical and creative thinking skills. Several teachers described that the approach has inspired them to teach in an innovative way despite the limited resources. In other words, they believe that the approach will not only promote students’ creativity but will also give teachers a chance to come up with creative lesson plans, such as using a toy car to teach concepts in mechanics and energy conversion, and using a Cartesian diver toy in teaching the concept of density.

The teachers were also made aware that environment values could also be developed through STEM projects. For example, in the toy solar car project, teachers themselves looked for recycled materials to make the chassis of their toy cars. Positive comments from the teachers are suggestive of how their students can be taught to value the environment and promote recycling. Another aspect of the findings is that teachers value the interdisciplinary nature within STEM projects. Such responses highlight the possibility of implementing STEM-PjBL activities in schools so that students can learn across disciplinary boundaries within the STEM subjects.

Despite the many positive views of teachers on the STEM-PjBL approach in teaching science, there are however some challenges that have been raised. One challenge highlighted is the amount of time that teachers need to carry out their projects. This challenge has also been described in the works of Straw et al. (2012), Johari et al. (2013). In addressing this challenge, findings from the views of the teachers suggest that it could be possible for the STEM-PjBL lessons to be carried out after school hours to ensure that teachers have enough time to complete teaching the science syllabus, and students have enough time to complete their projects. Another challenge highlighted by teachers in this study was on the limitation of resources and cost allocated for the projects. For example, in the toy solar car project, apart from using recycled materials, for the chassis of the cars, there is also a need to purchase the motor and solar panels. This is especially obvious when a large amount of materials are needed to cope with 40 to 50 students in a class. This challenge has also been described in the works of Wang et al. (2011), Straw et al. (2012) and Weber et al. (2013). In addressing this challenge, science teachers recommended that students and other stakeholders should take their own initiative in getting the necessary material for science projects, and conducting activities are in smaller groups.

The study also highlighted that the lack of STEM training could be a challenge for a teacher adopting a STEM-PjBL in his or her lessons. The work of Honey et al. (2014) highlights that ‘STEM’ is an integration of disciplines, and that knowing only the science or mathematics discipline alone may not be sufficient to execute a STEM-based lesson in the classroom. In order to adopt STEM-PjBL in science teaching, a teacher needs to be well equipped with not only the content knowledge in science or mathematics, but also the instructional skills in delivering science content through a STEM-PjBL (Ferry et al. 2005, and Walker et al. 2011). Wan et al. (2013) has brought up the idea of getting more support for STEM training for Malaysian teachers. The involvement of pre-service and in-service teacher participants in this workshop highlighted that it could be possible for them to be engaged in acquiring pedagogical content knowledge in teaching science through a STEM-PjBL approach.

Because the STEM-PjBL approach offers a different approach to conventional teaching approaches (such as didactic or stepped-through science experiments), training in STEM such as the one conducted in this study has allowed teachers to realize the need to plan STEM-PjBL lessons differently (in advance) especially for students who are not interested in Science. Also, because the STEM-PjBL approach is its interdisciplinary nature, it provides opportunities for teachers to plan on how students can learn across disciplinary boundaries in Mathematics, Physics, Science, Technology, Design and Engineering. Teachers can support students to bridge the gaps between separate learning areas through a single project. The positive views of teachers in this study mirrors those reported in earlier studies (Turgut 2008; Berry et al. 2012), which highlighted that a STEM-PjBL approach would enable students to gain experience in meeting up to problems that exist in the real world which are ill-defined, and ones that require students to draw knowledge from the STEM disciplines. Their views are also similar to the ones highlighted by Weber et al. (2013), in which teachers found that STEM projects act as catalysts to help students think critically and creatively in the STEM subject areas. These attributes such as being able to see links across subjects and being able to think critically and creatively in the STEM domains could contributes to a Malaysian student’s employability skills and marketability not just locally but also globally.

### Implications and recommendations

Findings from this study indicate that while considerable effort is necessary to put STEM-PjBL approaches into place, STEM-PjBL would provide practical, innovative curriculum development to support the Malaysian Educational Blueprint goals for 2013–2025. Findings from this study also revealed a number of gaps that would be needed to address in terms of training and support for teachers. This study has shown that several teachers have honestly expressed their lack of expertise in conducting STEM-PjBL approaches in their school science classes, one of the reasons being a possible reluctance to move away from conventional modes of teaching. This implies the need for educational institutions to develop more STEM-based training programs for classroom practitioners, particularly in the areas of lesson planning, instruction, STEM content, assessment techniques and fostering creative thinking skills. This could be achieved through on-going in-service STEM professional development programs. Findings also indicated that introducing five STEM-based activities at one time may overwhelm teachers. Introducing STEM-based approaches that use the latest effective teaching styles (such as the STEM-PjBL) a little bit at a time would be one way to address this challenge.

The research suggests the need to revise and re-structure the science and mathematics curriculum to produce students who are capable to think of Science, Technology, Engineering, and Mathematics in the broader terms, and not just as subjects they study in schools or just to pass examinations. There is a need to reform the Malaysian national curriculum to offer ways for students to link STEM subjects to solve real life/world problems. The STEM-PjBL approach as shown through this study can be a way to achieve this goal. Indeed, aspects of the STEM-PjBL approach could relatively easily be infused into the Malaysian national science and mathematics curriculum.

While this may be desirable, the researchers acknowledge that it is not that simple to design a multi-disciplinary project with wide coverage of STEM subjects. The researchers recommend that experts and STEM education researchers plan several multi-disciplinary projects that could present content across subjects such as Mathematics, General Science, Physics, Chemistry, Biology, Design, and Technology to students in schools. Students can then be offered one or two such projects each year, as they work towards meeting a certain curriculum requirement. This may be a practical way to prepare students to be capable of dealing with industrial-related problems when they enter the world of work.

A potential challenge that teachers raised is the amount of time needed to complete STEM projects in their science classes. The researchers found that it is important to provide sufficient time within or outside the curriculum so that the students can carry out their projects well. Also grades should be awarded for these projects based on the work and hours spent on planning and carrying them out. Credits could also be awarded for students who carry out some form of research in how content gained from their STEM projects subjects are applied in the real world.

Findings from this research also highlight an issue about infrastructure constraints. The study suggests that government should allocate sufficient budget to improve facilities and provide necessary equipment to furnish science laboratories to ensure that STEM-PjBL can be effectively achieved. Apart from the education ministry, funds could be contributed by private, STEM-related companies. Other stakeholders in education such as school staff, parents and universities could invite participation and collaboration from STEM-related companies, in view of building a future nation of STEM-trained students. This could not only develop the possibility of STEM experts and professionals to contribute to the Malaysian and global economy but could also place Malaysia’s science teaching at a higher level. The researchers believe that when this happens, there would be a positive reflection in Malaysian students’ performance in TIMSS and PISA results.

Finally, the study highlights a need for scholars to focus more strongly on raising teachers’ awareness of the interdisciplinary nature of STEM practices and to support the integration of STEM approaches in teaching practices. The Malaysian national curriculum may need to be re-vamped to enable this. Further studies should be carried out in the near future to gain more insights on constructing suitable models for Malaysian STEM education and monitoring these. Potential further research could usefully address our understanding of the obstacles faced by teachers in implementing STEM-PjBL approaches in classrooms perceptions of students toward the STEM projects, and teachers’ unbiased views of implementing STEM in their classrooms. Researchers will be required to set the standards for STEM education, produce instruments to assess the suitability of STEM-PjBL plans, and produce assessment tools in order to assess student’s competency in STEM.

Study findings further suggest that involvement of the various education stakeholders, namely teachers, the ministry, STEM-related agencies, institutes of higher learning such as universities, experts and scholars will all be required and valued on the journey to produce STEM-competent students.
